# Single nucleotide polymorphisms in obesity-related genes and all-cause and cause-specific mortality: a prospective cohort study

**DOI:** 10.1186/1471-2350-10-103

**Published:** 2009-10-09

**Authors:** Lisa Gallicchio, Howard H Chang, Dana K Christo, Lucy Thuita, Han Yao Huang, Paul Strickland, Ingo Ruczinski, Sandra Clipp, Kathy J Helzlsouer

**Affiliations:** 1 The Prevention and Research Center, The Weinberg Center for Women's Health & Medicine, Mercy Medical Center, 227 St Paul Place, 6th floor, Baltimore, Maryland, 21202, USA; 2 Department of Epidemiology, The Johns Hopkins Bloomberg School of Public Health, 615 N. Wolfe Street, Baltimore, Maryland, 21205, USA; 3 Department of Biostatistics, The Johns Hopkins Bloomberg School of Public Health, 615 N. Wolfe Street, Baltimore, Maryland, 21205, USA; 4 Department of Environmental Health Sciences, Johns Hopkins Bloomberg School of Public Health, 615 N. Wolfe Street, Baltimore, Maryland, 21205, USA

## Abstract

**Background:**

The aim of this study was to examine the associations between 16 specific single nucleotide polymorphisms (SNPs) in 8 obesity-related genes and overall and cause-specific mortality. We also examined the associations between the SNPs and body mass index (BMI) and change in BMI over time.

**Methods:**

Data were analyzed from 9,919 individuals who participated in two large community-based cohort studies conducted in Washington County, Maryland in 1974 (CLUE I) and 1989 (CLUE II). DNA from blood collected in 1989 was genotyped for 16 SNPs in 8 obesity-related genes: monoamine oxidase A (*MAOA*), lipoprotein lipase (*LPL*), paraoxonase 1 and 2 (*PON1 *and *PON2*), leptin receptor (*LEPR*), tumor necrosis factor-α (*TNFα*), and peroxisome proliferative activated receptor-γ and -δ (*PPARG *and *PPARD*). Data on height and weight in 1989 (CLUE II baseline) and at age 21 were collected from participants at the time of blood collection. All participants were followed from 1989 to the date of death or the end of follow-up in 2005. Cox proportional hazards regression was used to obtain the relative risk (RR) estimates and 95% confidence intervals (CI) for each SNP and mortality outcomes.

**Results:**

The results showed no patterns of association for the selected SNPs and the all-cause and cause-specific mortality outcomes, although statistically significant associations (p < 0.05) were observed between *PPARG *rs4684847 and all-cause mortality (CC: reference; CT: RR 0.99, 95% CI 0.89, 1.11; TT: RR 0.60, 95% CI 0.39, 0.93) and cancer-related mortality (CC: reference; CT: RR 1.01, 95% CI 0.82, 1.25; TT: RR 0.22, 95% CI 0.06, 0.90) and *TNFα *rs1799964 and cancer-related mortality (TT: reference; CT: RR 1.23, 95% CI 1.03, 1.47; CC: RR 0.83, 95% CI 0.54, 1.28). Additional analyses showed significant associations between SNPs in *LEPR *with BMI (rs1137101) and change in BMI over time (rs1045895 and rs1137101).

**Conclusion:**

Findings from this cohort study suggest that the selected SNPs are not associated with overall or cause-specific death, although several *LEPR *SNPs may be related to BMI and BMI change over time.

## Background

Epidemiologic studies from large, population-based cohorts have shown that obesity, defined by a body mass index (BMI) of 30 kg/m^2 ^or greater, is associated with an increased risk of mortality [1-6]. The biological plausibility of this association is well established, as it is known that excess body fat can lead to cardiovascular disease, type 2 diabetes mellitus, and cancer [7].

Individual behaviors, such as physical activity and eating habits, are known to play important roles in the development of obesity [7]. However, it has been hypothesized, and observed in some studies, that genetic factors may also be involved. These genetic factors include single nucleotide polymorphisms (SNPs) in genes that encode proteins involved in biological processes influencing body composition, including fat metabolism and adipocyte differentiation (peroxisome proliferator activated receptor γ (*PPARG*)) [8], hydrolyzation of triglycerides in both chylomicrons and very-low density lipoproteins (lipoprotein lipase (*LPL*)) [9], regulation of leptin, a protein involved in body weight regulation (leptin receptor (*LEPR*)) [10], prevention of low-density lipoprotein oxidation (paraoxonase 1 (*PON1*) and 2 (*PON2*)) [11], and regulation of monoamine levels (monoamine oxidase A (*MAOA*)) [12]. A recent review by Yang et al [13] reported that there are over 400 studies examining SNPs in these types of genes and obesity, with 22 of these genes found to be associated with obesity in at least five studies [13,14]. While these genes may be associated with obesity, little is known as to whether these genes also influence mortality.

We analyzed data from two community-based cohorts named CLUE I and CLUE II, established in Washington County, Maryland in 1974 and 1989, respectively, to examine the associations between SNPs in obesity-related genes and overall and cause-specific mortality. As a secondary aim, we also examined the associations between the genetic polymorphisms and BMI and change in BMI over time. For this study, genotyping was conducted on DNA extracted from blood samples collected in 1989 for 16 SNPs in the following 8 genes: *MAOA*, *LPL*, *PON1*, *PON2*, *LEPR*, *PPARG*, tumor necrosis factor-α (*TNFα*), and peroxisome proliferator activated receptor δ (*PPARD*). These genes were part of a larger group of genes that were chosen by study investigators prior to the start of project funding in 1999; they were selected because the metabolic processes they control were thought to be relevant to carcinogenesis as well as other disease processes, including death. SNPs within the selected genes were chosen because the minor allele frequency was estimated to be at least 5% among Caucasians and the polymorphisms were either known to be functional or were likely to alter function because they encoded for a nonsynonymous amino acid change or were located within the 5' or 3' untranslated region of the gene and thus could potentially alter mRNA stability.

## Methods

### Study Population

In 1974 and 1989, two cohorts named CLUE I and CLUE II, derived from the slogan *Give us a CLUE to Cancer*, were established in Washington County, Maryland in order to collect blood samples from as many individuals as possible for the prospective examination of factors involved in the development of cancer and cardiovascular disease. In addition to a blood sample donation at baseline for all participants, data were obtained using an interviewer-administered questionnaire on age, gender, marital status, education, height and weight (CLUE II only), weight at age 21, cigarette smoking, and medication and vitamin supplement use within the 48 hours prior to blood donation. Blood pressure was also measured and, in 1989, total cholesterol was assayed. For this analysis, BMI at age 21 and at CLUE II baseline was calculated based the information on weight collected at each time point and height reported at CLUE II baseline. Individuals who donated blood to both CLUE I and CLUE II constitute the Odyssey Cohort (N = 8,394) [15].

In addition to the Odyssey Cohort, a CLUE II subcohort was developed for case-cohort studies that would be conducted using the CLUE II cohort data. The subcohort was identified by taking a 10% age-stratified, random sample of CLUE II participants who donated a blood specimen in 1989 and were adult residents of Washington County, Maryland. Of the 2,460 participants identified for the subcohort, 807 were also in the Odyssey Cohort (i.e., they had participated in both CLUE I and CLUE II as described above). These two groups were combined for the current analysis (n = 10,047).

Of the participants in the Odyssey Cohort and the CLUE II subcohort, DNA was successfully extracted from the buffy coat samples of 9,960 individuals (99%). DNA from these participants was genotyped for polymorphisms in genes controlling biological processes such as obesity that have been associated with multiple diseases. For the study presented in this manuscript, Odyssey and subcohort participants who were missing data on all of the chosen SNPs were excluded from the analysis (n = 41). This study was approved by the Johns Hopkins Bloomberg School of Public Health's Institutional Review Board.

### Genotyping

SNPs in obesity-related genes analyzed in the present study were chosen because the minor allele frequency was estimated to be at least 5% among Caucasians and the polymorphisms were either known to be functional or were likely to alter function because they encoded for a nonsynonymous amino acid change or were located within the 5' or 3' untranslated region of the gene and thus could potentially alter mRNA stability. Descriptions and dbSNP identifiers of the polymorphisms in obesity-related pathways selected are shown on Additional file 1.

DNA extracted from the preserved buffy coat samples collected in 1989 were used for genotyping. Blood samples were centrifuged at 1500 g for 30 minutes at room temperature and were subsequently separated into plasma, buffy coat, and red blood cells and frozen at -70°C within 24 hours of collection. The buffy coat remained frozen until DNA extraction was performed. The DNA extraction procedures used the alkaline lysis method [16]. Following DNA isolation, DNA samples were resuspended in 10 mM Tris-HCl/1 mM EDTA (TE) and DNA concentration was adjusted to 100 μg/mL. Genotyping was performed using TaqMan technology by Celera Genomics Co. (Rockville, MD) for the SNPs with rs numbers 1800629, 4684847, 709158, and 1175543. Genotyping was performed by Applied Biosystems Inc. (Foster City, CA) for the SNPs with rs numbers 1801291, 316, 662, 7493, 12026, 7602, 1045895, 1137101, 2016520, 1801282, 1799724, and 1799964. Laboratory technicians were masked to disease status. The genotyping success rates of the selected polymorphisms ranged from 93% to 98%; 7,596 (76.6%) of the individuals in the analytic dataset had data on all 16 SNPs.

### Outcome assessment

All participants were followed from the date of blood draw to the date of death or the end of follow-up (June 20, 2005), whichever came first. In the CLUE cohorts, deaths are identified through daily searches of obituaries, cross-linking with death certificates for Washington County and through searches of the Social Security Administration search for individuals aged 65 or older and the National Death Index. Cause of death is ascertained from the underlying cause on Maryland State death certificates as coded by state nosologists. Of specific interest in this study were cancer deaths, for which the underlying cause was coded as ICD-9 140-239 or ICD-10 C00-C97, and cardiovascular disease deaths, for which the underlying cause was coded as ICD-9 390-459 or ICD-10 I00-I99. During the follow-up period, 2,159 deaths were documented in the Odyssey cohort and the CLUE II subcohort. Of these, 791 (36.6%) were cardiovascular deaths, 574 (26.6%) were cancer deaths, and 775 (35.9%) were deaths due to other causes.

Approximately 4% (n = 334) of the Odyssey cohort and the CLUE II subcohort participants were lost to follow-up. Since these individuals were not documented to have died during the follow-up period, they were considered alive at the end of follow-up and censored at June 20, 2005.

### Statistical analysis

The Hardy-Weinberg equilibrium for each SNP was tested using data from the entire study sample by a goodness-of-fit approach using STATA version 9. Because the *MAO *gene is linked to the X chromosome, Hardy-Weinberg equilibrium for this gene was tested for women only. Linkage disequilibrium was assessed using Haploview, Version 3.2 (Cambridge, MA).

Multiple imputation [17] was used to account for missing SNP data (range of missing data: 3 to 7%). Five independent complete datasets were constructed by predicting missing genotypes with decision trees that employed all variables relevant for the model building (other SNPs in linkage disequilibrium, demographic variables/covariates associated with the SNP, and the outcome). If no variables were associated with the SNP (including the outcome), imputation was based on the marginal distribution of the SNP with the missing data. Relative risk (RR) estimates were then obtained by combining estimates and standard errors from analyzing the five complete datasets. Cox proportional hazards regression was used to obtain the RR and 95% confidence interval (95% CI) for each SNP and mortality outcomes. For cause-specific death outcomes, all competing deaths were treated as censored observations at time of death. Genetic variations were coded by genotype with the reference category being the homozygote of the major allele. The statistical significance of the association between each genotype with respect to each outcome was examined using a chi-square goodness of fit test. All analyses were stratified by 10-year age groups to allow for a different baseline hazard for each 10-year age group; in addition, all RR estimates were age- and gender-adjusted. Other variables considered for adjustment included baseline BMI, change in BMI from age 21 to baseline, education (less than 12, 12, greater than 12 years), cigarette smoking status (never, former, current), systolic blood pressure, and diastolic blood pressure. Because the RR estimates did not change after multivariable adjustment or by using the imputation methods, only age- and gender-adjusted and stratified estimates using the non-imputed data are presented.

SNP-SNP interactions for all single-locus markers were examined using the imputed data by logic regression, an adaptive regression approach based on Boolean combinations of binary variables [18]. Each SNP was recorded in two binary variables that described the genotype.

Specifically, one variable was created for the presence of the dominant allele and one variable for the homozygote. For SNPs with low minor allele frequencies, the heterozygote and the less common homozygote were combined. Using a logistic link with death being the outcome, Boolean combinations of those binary predictors were examined as new possible covariates, adjusting for age and gender simultaneously. Permutation tests for model selection were carried out described in Ruczinski et al [19].

Further, to explore the potential for each SNP to be associated with premature death (all cause or cause-specific), Kaplan-Meier curves for the associations between each SNP and the mortality outcomes were examined. There were no indications that any of the three genotype curves for each SNP diverged early in the follow-up time period rather than later; therefore, it was decided that no further analyses for premature death would be considered or displayed in the results.

Additional analyses were conducted to examine the associations for the SNPs with baseline BMI and BMI change from age 21 to baseline adjusted for age and gender. For both analyses, the outcome variable was treated as a continuous outcome and analyzed with Normal regression. An analysis was also conducted to examine the risk of having a BMI ≥25 kg/m^2 ^at baseline (newly "overweight") associated with the investigated SNPs among those who had a BMI <25 kg/m^2 ^at age 21.

All analyses were performed using R version 2.6.0 (The R Project for Statistical Computing, ) unless otherwise specified. A two-sided P value of < 0.05 was considered statistically significant.

## Results

Characteristics of the sample are shown on Table 1. The mean age of participants at the time of blood draw was 53.1 years (standard deviation (SD) = 15.5); 99% of the participants were of Caucasian race. Approximately 62% of the sample was female and 16.5% were current smokers. The mean BMI of participants at the time of blood draw was 26.3 kg/m^2 ^(SD = 4.9); 19.3% had a BMI of ≥30 kg/m^2^. About a quarter of the participants were taking hypertension medications; 5.3% were taking medication for elevated cholesterol.

**Table 1:** Characteristics of study sample at 1989 blood draw

**Characteristic**	
N	9919
Mean age (years) and SD	53.1 (15.5)
Female (%)	61.5
Married (%)	73.0
	
Cigarette smoking status	
Never (%)	53.9
Former (%)	29.6
Current (%)	16.5
	
Mean education (years)	12
	
Treatment for hypertension	
Yes (%)	24.2
Mean systolic blood pressure (mmHg)	138.8
Mean diastolic blood pressure (mmHg)	82.7
No (%)	75.7
Mean systolic blood pressure (mmHg)	124.4
Mean diastolic blood pressure (mmHg)	78.0
	
Treatment for elevated cholesterol	
Yes (%)	5.3
No (%)	94.7
	
Mean cholesterol (mg/dL)	208.8
	
Mean body mass index (kg/m^2^)	26.3
	
Body mass index (kg/m^2^) by category	
<25.0 (%)	42.9
25.0-29.9 (%)	37.8
≥30.0 (%)	19.3

All of the polymorphisms examined were in Hardy-Weinberg equilibrium. The following SNPs were in strong linkage disequilibrium (D' > 90): *PON2 *Ser299Cys and *PON2 *Ala136Gly (D' = 99), *LEPR *rs7602 and *LEPR *rs1045895 (D' = 99), *TNFα *rs1799964 and *TNFα *rs1799724 (D' = 98), and *PPARG *rs709158 and *PPARG *rs1175543 (D' = 97).

In general, there were no observable patterns of association for the selected SNPs and the all-cause and cause-specific mortality outcomes (see Additional file 2, Additional file 3, and Additional file 4), although some individual SNPs showed statistically significant associations. As reported in a previous publication [20], the *PPARG *rs4684847 genotype was significantly associated with all-cause mortality, although, when stratified by gender, the association was statistically significant only among males (see Additional file 5, Additional file 6). Further, *PPARG *rs4684847 was significantly associated with cancer-related mortality; the decrease in risk associated with carrying two of the recessive alleles was observed among females only, although this risk estimate in females was not statistically significant. Additionally, *TNFα *rs1799964 was significantly associated with cancer-related mortality in the entire sample (*p = 0.03*); however, these was no evidence of a dose-response trend between number of variant alleles and the risk of cancer-related death. There were no statistically significant associations between the genotypes and cardiovascular-related mortality in the entire sample or among males and females separately. None of the associations reported above materially changed after adjustment for BMI or BMI change from age 21 to baseline, including the associations reported above. When the joint effects of the SNPs were considered, no statistically significant interactions were observed: the likelihoods of the interrogated SNP-SNP interactions could be explained by chance alone.

The associations for the selected SNPs with baseline BMI, BMI change from age 21 to baseline, and BMI ≥25 kg/m^2 ^at baseline among those with BMI <25 kg/m^2 ^at age 21 are reported on Table 2. The *LEPR *Gln223Arg SNP was significantly associated with BMI after adjustment for age and gender (*p *= 0.02); however, there was no dose-response relationship present between BMI and the number of variant alleles. The *LEPR *rs1045895 and *LEPR *Gln223Arg SNPs were significantly associated with change in BMI from age 21 to baseline such that, for both SNPs, the mean BMI change was significantly related to the number of variant alleles (*p *< 0.05). Finally, the *PPARG *Pro12Ala SNP was significantly associated with having a BMI ≥25 kg/m^2 ^at baseline among individuals with a BMI <25 kg/m^2 ^at age 21. Specifically, individuals who were <25 kg/m^2 ^at age 21 and who carried the Ala/Ala genotype were almost two times as likely to have a BMI ≥25 kg/m^2 ^at baseline compared to individuals who were <25 kg/m^2 ^at age 21 and carried the Pro/Pro genotype (OR = 1.91; 95% CI 1.28, 2.88). These associations did not differ by gender. There were no statistically significant associations between the other selected SNPs and BMI or change in BMI from age 21 to baseline among all participants or among males and females separately.

**Table 2:** Associations of obesity-related SNPs with BMI, change in BMI, and risk of becoming overweight

		**BMI (kg/m^2^) at baseline (1989)**		**BMI change from age 21 to baseline (1989)**		**BMI ≥25 kg/m^2 ^at baseline (1989) among those with BMI <25 kg/m^2 ^at age 21^a^**	
**Gene**	**rs #**	**Difference in mean^b^** **(95% CI)**	**p-value**	**Difference in mean^b^** **(95% CI)**	**p-value**	**Odds ratio^b^** **(95% CI)**	**p-value**

PPARG	4684847		0.36		0.57		0.20
CC		Reference		Reference		1.00 (reference)	
CT		0.18 (-0.07, 0.43)		-0.07 (-0.30, 0.15)		1.06 (0.95, 1.20)	
TT		0.17 (-0.67, 1.02)		0.30 (-0.45, 1.06)		1.35 (0.91, 2.01)	
PPARG	709158		0.88		0.59		0.86
AA		Reference		Reference		1.00 (reference)	
GA		0.05 (-0.16, 0.27)		0.09 (-0.10, 0.28)		0.97 (0.88, 1.07)	
GG		0.02 (-0.29, 0.33)		0.11 (-0.17, 0.39)		0.99 (0.86, 1.14)	
PPARG	1175543		0.99		0.52		0.79
AA		Reference		Reference		1.00 (reference)	
AG		-0.01 (-0.22, 0.20)		0.05 (-0.14, 0.24)		0.97 (0.88, 1.07)	
GG		-0.03 (-0.34, 0.29)		0.16 (-0.12, 0.44)		0.99 (0.85, 1.14)	
PPARG	1801282		0.69		0.70		0.01
CC		Reference		Reference		1.00 (reference)	
CG		0.09 (-0.16, 0.34)		-0.02 (-0.24, 0.20)		1.03 (0.92, 1.16)	
GG		0.23 (-0.6, 1.06)		0.31 (-0.44, 1.06)		1.91 (1.28, 2.88)	
PPARD	2016520		0.61		0.40		0.54
TT		Reference		Reference		1.00 (reference)	
CT		-0.09 (-0.31, 0.12)		-0.13 (-0.32, 0.06)		0.97 (0.88, 1.07)	
CC		0.11 (-0.42, 0.64)		-0.08 (-0.56, 0.39)		1.10 (0.86, 1.41)	
LPL	316		0.39		0.55		0.78
CC		Reference		Reference		1.00 (reference)	
AC		0.07 (-0.17, 0.31)		0.1 (-0.09, 0.29)		1.03 (0.94, 1.14)	
AA		0.53 (-0.28, 1.34)		0.01 (-0.28, 0.30)		1.00 (0.86, 1.16)	
PON1	662		0.61		0.59		0.75
TT		Reference		Reference		1.00 (reference)	
CT		-0.08 (-0.29, 0.12)		0.10 (-0.11, 0.32)		1.04 (0.93, 1.17)	
CC		-0.14 (-0.51, 0.22)		-0.12 (-0.85, 0.60)		0.96 (0.65, 1.40)	
LEPR	7602		0.47		0.71		0.34
GG		Reference		Reference		1.00 (reference)	
AG		-0.14 (-0.35, 0.08)		-0.06 (-0.24, 0.13)		0.93 (0.85, 1.02)	
AA		-0.05 (-0.55, 0.45)		0.07 (-0.26, 0.39)		0.98 (0.83, 1.16)	
LEPR	1045895		0.69		0.01		0.12
GG		Reference		Reference		1.00 (reference)	
AG		0.08 (-0.14, 0.30)		-0.28 (-0.47, -0.09)		0.93 (0.84, 1.03)	
AA		-0.02 (-0.31, 0.28)		-0.30 (-0.75, 0.15)		1.16 (0.92, 1.48)	
LEPR	1137101		0.02		0.04		0.39
AA		Reference		Reference		1.00 (reference)	
AG		-0.18 (-0.41, 0.05)		0.22 (0.03, 0.42)		1.07 (0.97, 1.19)	
GG		0.18 (-0.11, 0.46)		0.26 (0.00, 0.53)		1.05 (0.92, 1.21)	
PON2	7493		0.50		0.18		0.27
GG		Reference		Reference		1.00 (reference)	
CG		0.02 (-0.19, 0.23)		-0.17 (-0.38, 0.04)		0.94 (0.85, 1.05)	
CC		0.27 (-0.18, 0.72)		0.00 (-0.26, 0.25)		1.03 (0.90, 1.18)	
PON2	12026		0.59		0.64		0.05
CC		Reference		Reference		1.00 (reference)	
CG		0.03 (-0.18, 0.24)		0.08 (-0.10, 0.27)		1.08 (0.98, 1.19)	
GG		0.23 (-0.22, 0.68)		0.11 (-0.29, 0.51)		1.25 (1.02, 1.55)	
TNFα	1800629		0.27		0.56		0.07
GG		Reference		Reference		1.00 (reference)	
GA		0.15 (-0.07, 0.38)		0.09 (-0.09, 0.28)		1.08 (0.98, 1.19)	
AA		-0.22 (-0.82, 0.39)		0.13 (-0.27, 0.54)		1.23 (1.00, 1.51)	
TNFα	1799724		0.19		0.19		0.06
CC		Reference		Reference		1.00 (reference)	
CT		-0.24 (-0.50, 0.02)		0.19 (-0.01, 0.38)		1.11 (1.00, 1.23)	
TT		0.11 (-0.91, 1.13)		0.07 (-0.47, 0.62)		0.84 (0.63, 1.12)	
TNFα	1799964		0.06		0.31		0.87
TT		Reference		Reference		1.00 (reference)	
CT		0.22 (0.01, 0.43)		-0.18 (-0.42, 0.06)		1.01 (0.89, 1.14)	
CC		-0.16 (-0.61, 0.29)		0.11 (-0.81, 1.03)		0.88 (0.55, 1.40)	
MAOA (F)	1801291		0.45		0.30		0.82
CC		Reference		Reference		1.00 (reference)	
CT		0.16 (-0.13, 0.45)		0.10 (-0.09, 0.29)		1.02 (0.93, 1.13)	
TT		-0.10 (-0.61, 0.42)		-0.19 (-0.60, 0.21)		0.96 (0.78, 1.18)	
MAOA (M)	1801291		NA		NA		NA
CC		Reference		Reference		1.00 (reference)	
TT		-0.07 (-0.36, 0.22)		0.08 (-0.17, 0.33)		1.02 (0.90, 1.15)	

95% CI, 95% confidence interval; NA, not applicable

^a ^n = 7970 with self-reported BMI < 25 kg/m2 at age 21

^b ^Adjusted for age and gender

## Discussion

In this community-based cohort study, we examined the associations between 16 SNPs in 8 obesity-related genes and found no strong evidence that the selected SNPs were associated with overall or cause-specific mortality. These results are consistent with some, but not all, of the studies that make up the small body of literature examining SNPs and longevity. The most frequently studied SNP of those selected in this study is the *PON1 *Gln192Arg SNP; the reported findings for the association between this SNP and survival appear to differ by population. For example, the *PON1 *192Arg allele was found to be significantly more common in nonagenarians and/or centenarians versus centenarians offspring and controls in several studies conducted in Italy [21-23] (samples size of 308 centenarians and 579 controls in Bonafe *et al *[22] and 96 nonagenarians/centenarians and 173 controls in Marchegiani *et al *[23]), and was found to be associated with survival among 1,932 Danish individuals aged 47 to 93 [24]. In contrast, an association between this SNP and longevity was not observed in a sample of 592 Irish men and women [21], among American Caucasian long-living individuals (n = 749) and young controls (n = 355) [25], or in a cohort of 666 Danish subjects aged 85 years or older [26]. *PON1 *has been shown to be involved in the protection of LDL against lipid oxidation, and the functionality of the *PON1 *Gln192Arg SNP has been demonstrated, with the 192Gln/Gln genotype showing the most protection and the 192Arg/Arg showing the least [27]. The reasons for the inconsistent results regarding this SNP and longevity are unclear; however, most studies have been of small sample size, making the detection of weak associations difficult. Further, differing results may be the result of differences in environmental exposures that may alter expression of the genes in different populations [25,28].

This study also provided little evidence that the selected SNPs are associated with body mass or change in BMI over time, although the *LEPR *rs1045895 and *LEPR *Gln223Arg SNPs were found to be significantly associated with BMI change from age 21 to baseline. To our knowledge this is the first study to report on such associations; however, studies have examined the *LEPR *Gln223Arg SNP in relation to obesity and a recent meta-analysis showed no evidence of an association between the *LEPR *Gln223Arg SNP with the obesity outcome [29]. This report is not inconsistent with our results, as the *LEPR *Gln223Arg SNP was not associated with BMI or with the mortality outcomes. The *LEPR *rs1045895 SNP is in an intron region and was not associated with mortality; however, it may be linked to functional SNPs that play a role in a pathway leading to a gain in BMI over an individual's lifetime.

As the prevalence of overweight and obesity rises, there has been an increasing interest in the potential role of genetics in the development of obesity. Recent results from several genome wide association studies showed that SNPs in the fat mass and obesity gene (*FTO*), a gene not examined in this study, were associated with obesity among both children and adults, stimulating interest in the genetic origins of obesity [30-34]. Further, new findings from a population of 362,200 Danish men suggest that the minor A-allele in *FTO *rs9939609 is associated with an increased risk of mortality, although, interestingly, the association was observed independent of fatness (i.e. BMI, fat mass, and fat-BMI) [35]. Other new findings have shed light on a probable genetic origin of obesity; these findings include those reported in studies showing associations between BMI and/or other measures of body composition and SNPs in the catenin, beta like 1 gene (*CTNNBL1*) [36], the melanocortin-4 receptor (*MC4R*) [31,32,37-40] and the insulin-induced gene 2 (*INSIG2*) [36,41-43], among others. Additionally, SNPs in the genes investigated in this study, other than those measured, have been shown to be associated with obesity, body mass, and other cardiovascular risk factors. For example, in a study of 3,653 residents of Osaka, Japan, researchers showed the *LEPR *Arg109Lys SNP was associated with obesity such that individuals with the 109Lys/Lys genotype were at increased risk compared to those with the 109Arg/Arg genotype [44]. Investigating the associations between these SNPs and mortality would provide additional insight in to the long-term health effects of carrying specific obesity-related genotypes.

Obesity is a major public health concern. In a recent report, the International Obesity Taskforce estimated that, worldwide, there are a total of 1.1 billion adults are overweight, including 320 million who are obese [45]. Presently, the evidence points more towards the need for primary and secondary prevention efforts that include the incorporation of better health habits, such as eating right and exercising, and away from the identification of at-risk individuals through genetic screening. However, future research should focus on explore whole genome scanning approaches to find clues for multiple pathway interactions that may lead towards obesity and mortality.

## Conclusion

Findings from this cohort study suggest that the selected SNPs are not associated with overall or cause-specific death, although several *LEPR *SNPs may be related to BMI and BMI change over time. Focus should be primarily on the incorporation of better health habits to prevent obesity and not genetic screening.

## Abbreviations

95% CI: 95% confidence interval; BMI: body mass index; CTNNBL1: catenin, beta like 1 gene; FTO: fat mass and obesity gene; INSIG2: insulin-induced gene 2; LEPR: leptin receptor; LPL: lipoprotein lipase; MAOA: monoamine oxidase A; MC4R: melanocortin-4 receptor; PON1: paraoxonase 1; PON2: paraoxonase 2; PPAR: peroxisome proliferator-activated receptor; RR: relative risk; SNP: single nucleotide polymorphism; TNF: tumor necrosis factor.

## Competing interests

The authors declare that they have no competing interests.

## Authors' contributions

LG: data analysis, drafting of the manuscript; HC: data analysis, reviewing and revising the manuscript; DC: acquisition and analysis of the data, reviewing and revising the manuscript; LT: acquisition and analysis of the data, reviewing and revising the manuscript; HYH: design and coordination of the study, reviewing the manuscript; IR: data analysis, interpretation of the data, drafting of the manuscript; PS: design and coordination of the study; SC: design and coordination of the study, acquisition of the data; KH: conceived of the study, design and coordination of the study; drafting of the manuscript.

All authors read and approved the final manuscript.

## Pre-publication history

The pre-publication history for this paper can be accessed here:



## Supplementary Material

Additional file 1 **Description of single nucleotide polymorphisms in obesity-related genes investigated in the Odyssey Cohort.** Click here for file

Additional file 2 **Age- and gender-adjusted associations between SNPs in obesity-related genes and mortality.** Click here for file

Additional file 3 **Associations between obesity-related SNPs and cardiovascular mortality adjusted for age and gender.** Click here for file

Additional file 4 **Associations between obesity-related SNPs and cancer mortality adjusted for age and gender.** Click here for file

Additional file 5 **Associations between obesity-related SNPs and mortality adjusted for age among males only.** Click here for file

Additional file 6 **Associations between obesity-related SNPs and mortality adjusted for age among females only.** Click here for file
