# Supplementary material for: Single nucleotide polymorphisms in obesity-related genes and all-cause and cause-specific mortality: a prospective cohort study
Source: BMC Med Genet. 2009 Oct 9;10:103. doi: 10.1186/1471-2350-10-103 (PMC2763854; doi:10.1186/1471-2350-10-103)
Supplement: Additional file 1 — Description of single nucleotide polymorphisms in obesity-related genes investigated in the Odyssey Cohort. [file 1471-2350-10-103-S1.DOC]

**Additional Table 1**

*Description of single nucleotide polymorphisms in obesity-related genes investigated in the Odyssey Cohort*

| **Gene** | **Gene symbol** | **dbSNP**  **rs #** | **snp** | **Amino Acid Substitution** | **Frequency of minor allele** | **Genotyping Success Rate** |
| --- | --- | --- | --- | --- | --- | --- |
|  |  |  |  |  |  |  |
| Monoamine oxidase A | MAOA | 1801291 |  |  | 0.28 | 0.97 |
| Lipoprotein lipase | LPL | 316 | Ex8+25C>A | Thr338Thr | 0.12 | 0.97 |
| Paraoxonase1 | PON1 | 662 | Ex6+78A>G | Gln192Arg | 0.29 | 0.96 |
| Paraoxonase2 | PON2 | 7493 | Ex9+26C>G | Ser299Cys | 0.23 | 0.97 |
| Paraoxonase2 | PON2 | 12026 | Ex5-52C>G | Ala136Gly | 0.23 | 0.96 |
| Leptin receptor | LEPR | 7602 | IVS2+6890A>G |  | 0.20 | 0.96 |
| Leptin receptor | LEPR | 1045895 | IVS2+6920G>A |  | 0.40 | 0.96 |
| Leptin receptor | LEPR | 1137101 | Ex6-36A>G | Gln223Arg | 0.45 | 0.93 |
| Tumor necrosis factor-α | TNFα | 1800629 | -487A>G |  | 0.16 | 0.95 |
| Tumor necrosis factor-α | TNFα | 1799724 | -1036T>C |  | 0.09 | 0.98 |
| Tumor necrosis factor-α | TNFα | 1799964 | -1210T>C |  | 0.22 | 0.97 |
| Peroxisome proliferative activated receptor-γ | PPARG | 4684847 | IVS2-6622C>T |  | 0.11 | 0.95 |
| Peroxisome proliferative activated receptor-γ | PPARG | 709158 | IVS9+4523A>G |  | 0.36 | 0.95 |
| Peroxisome proliferative activated receptor-γ | PPARG | 1175543 | IVS9+7780A>G |  | 0.36 | 0.95 |
| Peroxisome proliferative activated receptor-γ | PPARG | 1801282 | Ex4-49C>G | Pro12Ala | 0.11 | 0.96 |
| Peroxisome proliferative activated receptor-δ | PPARD | 2016520 | Ex4+15C>T |  | 0.19 | 0.96 |
