# Supplementary material for: Single nucleotide polymorphisms in obesity-related genes and all-cause and cause-specific mortality: a prospective cohort study
Source: BMC Med Genet. 2009 Oct 9;10:103. doi: 10.1186/1471-2350-10-103 (PMC2763854; doi:10.1186/1471-2350-10-103)
Supplement: Additional file 2 — Age- and gender-adjusted associations between SNPs in obesity-related genes and mortality. [file 1471-2350-10-103-S2.DOC]

**Additional Table 2**

*Age- and gender-adjusted* associations between SNPs in obesity-related genes and mortality

|  |  | All-cause mortality | | | |  | Cardiovascular mortality | | | |  | Cancer mortality | | | |
| --- | --- | --- | --- | --- | --- | --- | --- | --- | --- | --- | --- | --- | --- | --- | --- |
| Gene | rs # | n cases | person years | Adjusted RR  (95% CI) | p-value |  | n cases | person years | Adjusted RR  (95% CI) | p-value |  | n cases | person years | Adjusted RR  (95% CI) | p-value |
|  |  |  |  |  |  |  |  |  |  |  |  |  |  |  |  |
| PPARG | 4684847 |  |  |  | 0.04 |  |  |  |  | 0.69 |  |  |  |  | 0.02 |
| CC |  | 1613 | 107018 | 1.00 (reference) |  |  | 592 | 107018 | 1.00 (reference) |  |  | 428 | 107018 | 1.00 (reference) |  |
| CT |  | 401 | 26655 | 0.99 (0.89, 1.11) |  |  | 143 | 26655 | 0.98 (0.81, 1.17) |  |  | 110 | 26655 | 1.01 (0.82, 1.25) |  |
| TT |  | 21 | 1996 | 0.60 (0.39, 0.93) |  |  | 10 | 1996 | 0.77 (0.41, 1.45) |  |  | 2 | 1996 | 0.22 (0.06, 0.90) |  |
| PPARG | 709158 |  |  |  | 0.43 |  |  |  |  | 0.06 |  |  |  |  | 0.98 |
| AA |  | 834 | 56979 | 1.00 (reference) |  |  | 324 | 56979 | 1.00 (reference) |  |  | 224 | 56979 | 1.00 (reference) |  |
| GA |  | 927 | 61835 | 0.98 (0.89, 1.08) |  |  | 331 | 61835 | 0.89 (0.77, 1.04) |  |  | 248 | 61835 | 0.99 (0.83, 1.19) |  |
| GG |  | 267 | 17887 | 0.91 (0.80, 1.05) |  |  | 89 | 17887 | 0.77 (0.61, 0.97) |  |  | 74 | 17887 | 0.97 (0.75, 1.27) |  |
| PPARG | 1175543 |  |  |  | 0.50 |  |  |  |  | 0.06 |  |  |  |  | 0.93 |
| AA |  | 845 | 57226 | 1.00 (reference) |  |  | 325 | 57226 | 1.00 (reference) |  |  | 223 | 57226 | 1.00 (reference) |  |
| AG |  | 928 | 61474 | 0.99 (0.91, 1.09) |  |  | 331 | 61474 | 0.92 (0.79, 1.07) |  |  | 250 | 61474 | 1.02 (0.85, 1.22) |  |
| GG |  | 268 | 17853 | 0.92 (0.81, 1.06) |  |  | 86 | 17853 | 0.76 (0.60, 0.96) |  |  | 72 | 17853 | 0.97 (0.74, 1.26) |  |
| PPARG | 1801282 |  |  |  | 0.21 |  |  |  |  | 0.77 |  |  |  |  | 0.57 |
| CC |  | 1596 | 108306 | 1.00 (reference) |  |  | 583 | 108306 | 1.00 (reference) |  |  | 418 | 108306 | 1.00 (reference) |  |
| CG |  | 392 | 26945 | 0.98 (0.88, 1.10) |  |  | 136 | 26945 | 0.93 (0.78, 1.13) |  |  | 111 | 26945 | 1.06 (0.86, 1.30) |  |
| GG |  | 24 | 2050 | 0.71 (0.47, 1.06) |  |  | 12 | 2050 | 0.95 (0.54, 1.69) |  |  | 6 | 2050 | 0.71 (0.32, 1.58) |  |
| PPARD | 2016520 |  |  |  | 0.71 |  |  |  |  | 0.95 |  |  |  |  | 0.87 |
| TT |  | 1301 | 89337 | 1.00 (reference) |  |  | 479 | 89337 | 1.00 (reference) |  |  | 344 | 89337 | 1.00 (reference) |  |
| CT |  | 631 | 42802 | 1.02 (0.93, 1.12) |  |  | 226 | 42802 | 0.99 (0.84, 1.16) |  |  | 169 | 42802 | 1.03 (0.86, 1.24) |  |
| CC |  | 73 | 5007 | 0.93 (0.73, 1.17) |  |  | 29 | 5007 | 0.94 (0.65, 1.38) |  |  | 18 | 5007 | 0.92 (0.57, 1.48) |  |
| LPL | 316 |  |  |  | 0.37 |  |  |  |  | 0.85 |  |  |  |  | 0.36 |
| CC |  | 1625 | 107966 | 1.00 (reference) |  |  | 591 | 107966 | 1.00 (reference) |  |  | 424 | 107966 | 1.00 (reference) |  |
| AC |  | 399 | 28843 | 0.96 (0.86, 1.08) |  |  | 143 | 28843 | 0.96 (0.80, 1.15) |  |  | 113 | 28843 | 1.04 (0.84, 1.28) |  |
| AA |  | 26 | 2085 | 0.78 (0.53, 1.15) |  |  | 13 | 2085 | 1.10 (0.63, 1.90) |  |  | 5 | 2085 | 0.57 (0.24, 1.38) |  |
| PON1 | 662 |  |  |  | 0.28 |  |  |  |  | 0.90 |  |  |  |  | 0.78 |
| TT |  | 1010 | 69109 | 1.00 (reference) |  |  | 374 | 69109 | 1.00 (reference) |  |  | 264 | 69109 | 1.00 (reference) |  |
| CT |  | 831 | 56320 | 1.04 (0.94, 1.14) |  |  | 304 | 56320 | 1.03 (0.89, 1.20) |  |  | 227 | 56320 | 1.06 (0.89, 1.26) |  |
| CC |  | 186 | 11764 | 1.13 (0.97, 1.33) |  |  | 62 | 11764 | 1.04 (0.79, 1.36) |  |  | 48 | 11764 | 1.08 (0.79, 1.47) |  |
| LEPR | 7602 |  |  |  | 0.75 |  |  |  |  | 0.52 |  |  |  |  | 0.78 |
| GG |  | 1303 | 87708 | 1.00 (reference) |  |  | 476 | 87708 | 1.00 (reference) |  |  | 336 | 87708 | 1.00 (reference) |  |
| AG |  | 618 | 43096 | 1.02 (0.92, 1.12) |  |  | 222 | 43096 | 1.02 (0.87, 1.19) |  |  | 171 | 43096 | 1.07 (0.89, 1.28) |  |
| AA |  | 87 | 5505 | 0.93 (0.75, 1.16) |  |  | 28 | 5505 | 0.81 (0.56, 1.19) |  |  | 25 | 5505 | 1.07 (0.71, 1.60) |  |
| LEPR | 1045895 |  |  |  | 0.48 |  |  |  |  | 0.82 |  |  |  |  | 0.51 |
| GG |  | 731 | 50177 | 1.00 (reference) |  |  | 278 | 50177 | 1.00 (reference) |  |  | 197 | 50177 | 1.00 (reference) |  |
| AG |  | 938 | 65433 | 1.01 (0.91, 1.11) |  |  | 336 | 65433 | 0.96 (0.82, 1.12) |  |  | 243 | 65433 | 0.95 (0.79, 1.15) |  |
| AA |  | 348 | 21651 | 1.08 (0.95, 1.22) |  |  | 117 | 21651 | 0.94 (0.76, 1.17) |  |  | 95 | 21651 | 1.10 (0.86, 1.40) |  |
| LEPR | 1137101 |  |  |  | 0.46 |  |  |  |  | 0.31 |  |  |  |  | 0.69 |
| AA |  | 583 | 39704 | 1.00 (reference) |  |  | 204 | 39704 | 1.00 (reference) |  |  | 156 | 39704 | 1.00 (reference) |  |
| AG |  | 931 | 65547 | 0.94 (0.85, 1.05) |  |  | 338 | 65547 | 0.97 (0.81, 1.15) |  |  | 240 | 65547 | 0.92 (0.76, 1.13) |  |
| GG |  | 406 | 27696 | 0.99 (0.88, 1.13) |  |  | 163 | 27696 | 1.12 (0.91, 1.38) |  |  | 106 | 27696 | 0.99 (0.78, 1.27) |  |
| PON2 | 7493 |  |  |  | 0.54 |  |  |  |  | 0.77 |  |  |  |  | 0.49 |
| GG |  | 1186 | 80496 | 1.00 (reference) |  |  | 432 | 80496 | 1.00 (reference) |  |  | 307 | 80496 | 1.00 (reference) |  |
| CG |  | 722 | 50065 | 1.03 (0.93, 1.12) |  |  | 270 | 50065 | 1.06 (0.91, 1.23) |  |  | 192 | 50065 | 1.04 (0.87, 1.25) |  |
| CC |  | 112 | 7218 | 1.11 (0.92, 1.35) |  |  | 37 | 7218 | 1.05 (0.75, 1.46) |  |  | 34 | 7218 | 1.24 (0.87, 1.77) |  |
| PON2 | 12026 |  |  |  | 0.64 |  |  |  |  | 0.77 |  |  |  |  | 0.57 |
| CC |  | 1184 | 80423 | 1.00 (reference) |  |  | 431 | 80423 | 1.00 (reference) |  |  | 307 | 80423 | 1.00 (reference) |  |
| CG |  | 709 | 49445 | 1.03 (0.94, 1.13) |  |  | 263 | 49445 | 1.06 (0.91, 1.23) |  |  | 189 | 49445 | 1.05 (0.87, 1.26) |  |
| GG |  | 109 | 7223 | 1.08 (0.89, 1.32) |  |  | 37 | 7223 | 1.05 (0.75, 1.47) |  |  | 33 | 7223 | 1.21 (0.85, 1.73) |  |
| TNFα | 1800629 |  |  |  | 0.12 |  |  |  |  | 0.31 |  |  |  |  | 0.23 |
| GG |  | 1442 | 95098 | 1.00 (reference) |  |  | 535 | 95098 | 1.00 (reference) |  |  | 378 | 95098 | 1.00 (reference) |  |
| GA |  | 539 | 37355 | 0.95 (0.86, 1.05) |  |  | 184 | 37355 | 0.88 (0.74, 1.04) |  |  | 155 | 37355 | 1.05 (0.87, 1.26) |  |
| AA |  | 48 | 3816 | 0.76 (0.57, 1.02) |  |  | 23 | 3816 | 0.97 (0.64, 1.47) |  |  | 10 | 3816 | 0.62 (0.33, 1.16) |  |
| TNFα | 1799724 |  |  |  | 0.09 |  |  |  |  | 0.49 |  |  |  |  | 0.34 |
| CC |  | 1724 | 114931 | 1.00 (reference) |  |  | 621 | 114931 | 1.00 (reference) |  |  | 457 | 114931 | 1.00 (reference) |  |
| CT |  | 316 | 22922 | 0.89 (0.79, 1.01) |  |  | 123 | 22922 | 0.95 (0.78, 1.15) |  |  | 80 | 22922 | 0.86 (0.68, 1.10) |  |
| TT |  | 11 | 1339 | 0.72 (0.40, 1.29) |  |  | 3 | 1339 | 0.57 (0.18, 1.76) |  |  | 6 | 1339 | 1.39 (0.62, 3.11) |  |
| TNFα | 1799964 |  |  |  | 0.24 |  |  |  |  | 0.57 |  |  |  |  | 0.03 |
| TT |  | 1216 | 85355 | 1.00 (reference) |  |  | 469 | 85355 | 1.00 (reference) |  |  | 307 | 85355 | 1.00 (reference) |  |
| CT |  | 717 | 46079 | 1.08 (0.98, 1.18) |  |  | 239 | 46079 | 0.93 (0.80, 1.09) |  |  | 206 | 46079 | 1.23 (1.03, 1.47) |  |
| CC |  | 105 | 7096 | 0.98 (0.80, 1.20) |  |  | 37 | 7096 | 0.89 (0.64, 1.25) |  |  | 22 | 7096 | 0.83 (0.54, 1.28) |  |
| MAOA (F)^a^ | 1801291 |  |  |  | 0.76 |  |  |  |  | 0.33 |  |  |  |  | 0.49 |
| CC |  | 593 | 45445 | 1.00 (reference) |  |  | 213 | 45445 | 1.00 (reference) |  |  | 157 | 45445 | 1.00 (reference) |  |
| CT |  | 448 | 34211 | 0.99 (0.87, 1.12) |  |  | 159 | 34211 | 0.96 (0.78, 1.18) |  |  | 120 | 34211 | 1.01 (0.80, 1.29) |  |
| TT |  | 94 | 7006 | 1.08 (0.87, 1.34) |  |  | 39 | 7006 | 1.26 (0.90, 1.78) |  |  | 18 | 7006 | 0.76 (0.47, 1.24) |  |
| MAOA (M)^a^ | 1801291 |  |  |  | NA |  |  |  |  | NA |  |  |  |  | NA |
| CC |  | 670 | 37536 | 1.00 (reference) |  |  | 249 | 37536 | 1.00 (reference) |  |  | 169 | 37536 | 1.00 (reference) |  |
| TT |  | 235 | 14134 | 0.99 (0.85, 1.15) |  |  | 79 | 14134 | 0.90 (0.70, 1.16) |  |  | 77 | 14134 | 1.27 (0.97, 1.66) |  |

95% CI, 95% confidence interval; NA, not applicable; RR, relative risk

^a^ analyses were stratified by and not adjusted for gender for MAOA SNP
